# Androgen deprivation induces neuroendocrine phenotypes in prostate cancer cells through CREB1/EZH2-mediated downregulation of REST

**DOI:** 10.21203/rs.3.rs-3270539/v1

**Published:** 2023-10-04

**Authors:** Wenliang Li, Dayong Zheng, Yan Zhang, Sukjin Yang, Ning Su, Michael Bakhoum, Guoliang Zhang, Samira Naderinezhad, Zhengmei Mao, Zheng Wang, Ting Zhou

**Affiliations:** The University of Texas Health Science Center at Houston; Integrated Hospital of Traditional Chinese Medicine, Southern Medical University; The University of Texas Health Science Center at Houston; The University of Texas Health Science Center at Houston; The University of Texas Health Science Center at Houston; The University of Texas Health Science Center at Houston; Shanghai Sixth People’s Hospital, Shanghai Jiaotong University; Brown Foundation Institute of Molecular Medicine; The University of Texas Health Science Center at Houston; The University of Texas Health Science Center at Houston; The Brown Foundation Institute of Molecular Medicine, The University of Texas Health Science Center at Houston

## Abstract

Although effective initially, prolonged androgen deprivation therapy (ADT) promotes neuroendocrine differentiation (NED) and prostate cancer (PCa) progression. It is incompletely understood how ADT transcriptionally induces NE genes in PCa cells. CREB1 and REST are known to positively and negatively regulate neuronal gene expression in the brain, respectively. No direct link between these two master neuronal regulators has been elucidated in the NED of PCa. We show that REST mRNA is downregulated in NEPC cell and mouse models, as well as in patient samples. Phenotypically, REST overexpression increases ADT sensitivity, represses NE genes, inhibits colony formation in culture, and xenograft tumor growth of PCa cells. As expected, ADT downregulates REST in PCa cells in culture and in mouse xenografts. Interestingly, CREB1 signaling represses REST expression. In studying the largely unclear mechanism underlying transcriptional repression of REST by ADT, we found that REST is a direct target of EZH2 epigenetic repression. Finally, genetic rescue experiments demonstrated that ADT induces NED through EZH2’s repression of REST, which is enhanced by ADT-activated CREB signaling. In summary, our study has revealed a key pathway underlying NE gene upregulation by ADT, as well as established novel relationships between CREB1 and REST, and between EZH2 and REST, which may also have implications in other cancer types and in neurobiology.

## Introduction

Prostate cancer stands as the second most prevalent cancer and the second leading contributor to cancer-related mortality among men in the United States. The year 2022 witnessed an estimated 34,500 fatalities due to prostate cancer in the United States alone, as reported by the American Cancer Society’s statistics. Androgen deprivation therapies (ADT) focusing on the androgen receptor (AR) remain the cornerstone treatments for prostate cancer (PCa). Though ADT initially displays effectiveness, the majority of tumors inevitably experience recurrence and evolve into castration-resistant prostate cancer (CRPC). The process of lineage plasticity has gained prominence as a pivotal mechanism driving the progression of CRPC^1–3^.

Approximately 20% of fatal CRPC cases exhibit a significant presence of neuroendocrine-like tumor cells, referred to as treatment-related neuroendocrine prostate cancer (t-NEPC or CRPC-NE, herein termed NEPC)^4–6^. These NEPC cells are thought to originate, at least partially, from neuroendocrine differentiation (NED) of adenocarcinoma cells^7, 8^. Key attributes of NEPC cells encompass the loss of androgen receptor signaling, resilience against ADT, and heightened expression of neuroendocrine markers, including neuron-specific enolase (ENO2), synaptophysin (SYP), chromogranin A (CHGA), and chromogranin B (CHGB)^4, 9–11^. Nonetheless, the intricate mechanisms governing the induction of NE markers by ADT remain incompletely elucidated.

CREB1 and REST are two established master activator and repressor of neuronal genes in the brain, respectively^12–14^. In epithelial cancer cells, REST has been reported to act as a tumor suppressor^15^. REST represses NE gene expression in prostate and lung cancer cells^16–19^. We and others have demonstrated that activation of CREB1 signaling is critical for ADT-induction of NE markers^20–23^. It is still unclear how CREB activation induces NE markers, and there has been no direct link established between CREB1 and REST in the context of ADT-induced NED.

Polycomb repressive complex 2 (PRC2) represses gene transcription by catalyzing methylations on histone H3 lysine 27 (H3K27me)^24–27^, which are repressive histone marks. The major enzyme for catalyzing histone H3K27 methylations is EZH2 (Enhanced Zeste Homolog 2)^28–30^. EZH2 is overexpressed in several solid tumors, such as prostate, breast, and lung cancers^29, 31–38^. EZH2 expression and its PRC2 activity are particularly high in NEPC^39–41^. In prostate cancer, EZH2 is known to collaborate with AR in promoting the progression of AR-positive CRPCs through both H3K27me3-dependent and independent mechanisms^42, 43^. On the other hand, how EZH2 promotes the progression of AR-negative NEPC remains an open question^41, 44^.

REST and EZH2 are partners in transcriptional repression in mammalian cells^45–47^. REST has been shown to physically interact with EZH2, which can lead to the recruitment of PRC2 complex to repress REST targets, such as neuronal genes^48–50^. In addition to recruitment by REST, EZH2 has been shown to methylate REST, which stabilizes REST when bound to the RE1 sites of target genes^51^.

Previously, we reported that CREB1 signaling acts through EZH2’s PRC activity to induce NE markers^20^. However, the critical link between ADT-CREB1-EZH2 pathway and NE induction is still missing. As an epigenetic repressor in this NED context, as we and others have shown^20, 40^, EZH2 is expected to repress some transcription regulators that in turn suppress NE marker expression.

To better understand the mechanisms of NED in prostate cancer cells, here we investigated the links between ADT, CREB1, EZH2, and REST. We found that CREB1 activation leads to the downregulation of REST, and ADT induces NED through the CREB1/EZH2/REST pathways. Intriguingly, REST itself is an epigenetic target of EZH2 in NEPC cells. Our study has thus provided critical new information regarding how ADT, CREB1, and EZH2 induce NED, and also revealed a direct repression of REST by CREB1 signaling and by EZH2’s epigenetic regulation.

## Results

### REST is downregulated in NEPC.

**Figure 1:**
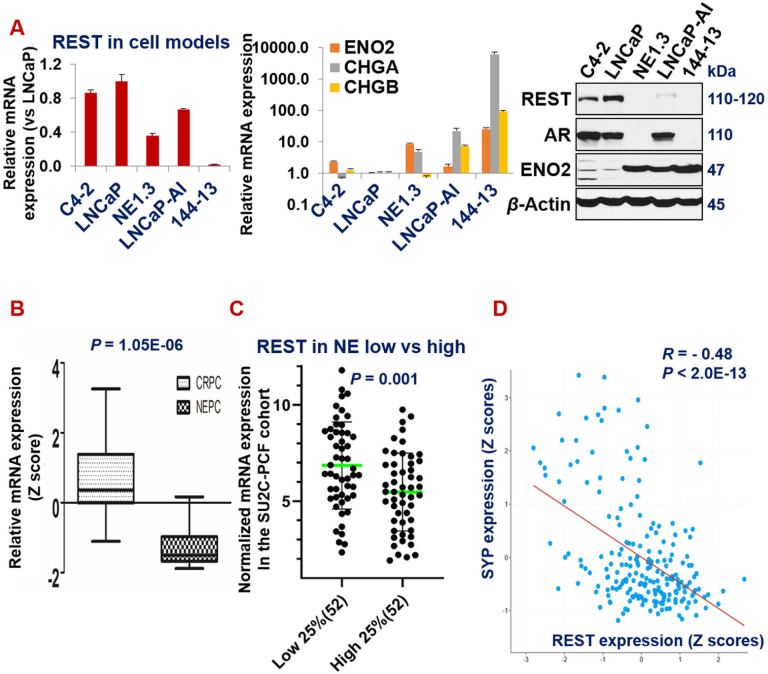
REST is downregulated in NEPC. (**A**) qPCR and Western blotting for indicated genes and proteins in a NEPC cell line 144-13, ADPC line LNCaP, and LNCaP-derived cell lines C4-2, NE1.3 and LNCaP-AI. Y-axis shows relative fold changes in mRNA expression, normalized to GAPDH. Error bars in PCR results represent standard deviation (s.d) of duplicate experiments. Beta actin was examined as the loading control in Western blotting. (**B**) REST mRNA is significantly lower in NEPC (CRPC-NE) than in adenocarcinoma CRPC (CRPC-adeno) (Beltran_NM2016)^39^. (**C**) REST mRNA in the top 25% NE-higher samples versus the bottom 25% NE-low samples of CRPC in SU2C-PCF dataset^53^. (**D**) Expression of NE marker SYP negatively correlates with that of REST in SU2C-PCF dataset. REST correlations for additional NE markers (ENO2, CHGA, CHGB, and TUBB3) are presented in Supplementary Fig. 1.

To determine the expression patterns of REST in adenocarcinoma and neuroendocrine prostate cancer (NEPC), we analyzed its expression patterns in a panel of ADPC (androgen-dependent prostate cancer) and NEPC cell lines, patient-derived xenografts (PDX), and patient samples. In line with the literature, REST mRNA and protein levels are lower, while NE markers are higher, in NE + PCa cell lines NE1.3, LNCaP-AI, and 144 − 13 than in NE- cell lines C4-2 and LNCaP (Fig. 1A). -Similar expression patterns were found in prostate cancer patient samples. Upon examining the Beltran_NM2016 RNA-seq dataset^39^, we found that REST is significantly lower, while NE markers are significantly higher, in 15 NEPC samples compared to 34 CRPC-adenocarcinoma samples (Fig. 1C, P = 1.05E-06). In another well-cited prostate cancer dataset (SU2C-PCF_PCa^53^), based on the NE scores provided in the dataset, we compared the cases in NE score top 25% versus those in the NE score bottom 25%. As shown in Fig. 1D, REST mRNA expression is significantly lower in the top 25% samples (P = 0.001). As expected, REST expression negatively correlates with NE markers in prostate cancer patient samples (Fig. 1E and Supplementary Fig. S1). Overall, these results confirm that REST expression is downregulated in NEPC.

### REST inhibits NE marker expression, ADT resistance, and tumor progression.

**Figure 2:**
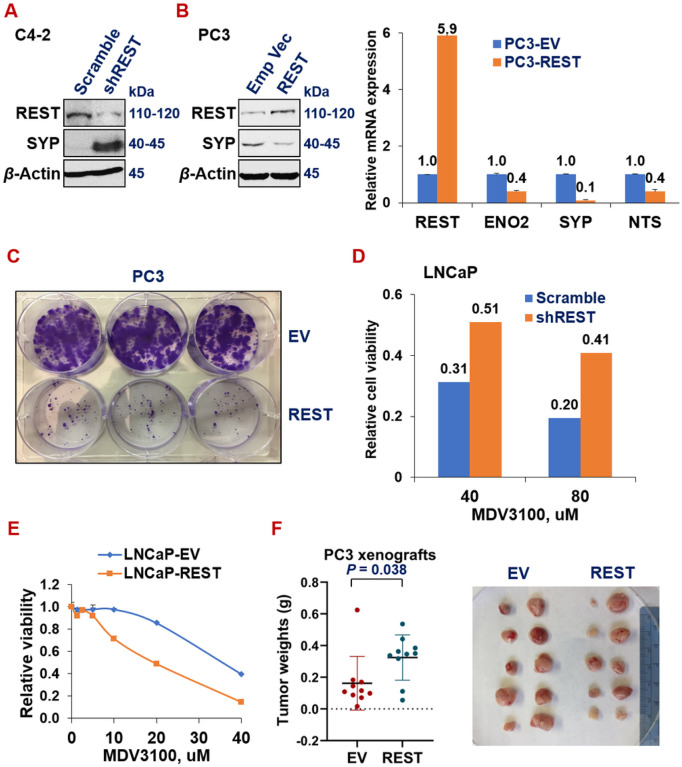
REST inhibits NE marker expression, ADT resistance and tumor progression. (**A**) Western blotting for REST and NE marker SYP in NE- C4-2 cells upon REST silencing and in NE+ PC3 cells upon overexpressing REST cDNA. (**B**)qPCR of additional NE markers in PC3 cells upon overexpressing REST cDNA. Y-axis shows relative fold changes in mRNA expression, normalized to GAPDH. Error bars in PCR results represent standard deviation (s.d) of duplicate experiments. (**C**) Colony formation assay of PC3 cells carrying empty vector (EV) or REST cDNA. The two cell lines were seeded in triplicates in a 6-well plate, at 1,000 cells/well and cultured for 14 days, followed by staining with crystal violet. (**D**) Viability and proliferation of LNCaP cells expressing either scramble shRNA or shREST, upon treatments of 40uM or 80uM of ADT drug MDV3100 for 3 days. (**E**) Cell viability and proliferation of LNCaP cells carrying either empty vector or REST cDNA, upon treating with a series doses of ADT drug MDV3100 for 3 days. Y-axis shows relative cell viability and proliferation, normalized to DMSO control. The experiment was carried out in quadruplicates each time, twice with similar results. (**F**)Subcutaneous xenograft tumor growth of PC3 cells carrying either EV or REST cDNA in NOD/SCID male mice. Left: tumor weights at euthanization (P=0.038) 8 weeks after implantation. Right: images of the xenograft tumors at euthanization (5 mice for each cell line, n=10 tumors).

To investigate the role of REST in prostate cancer progression, we modulated REST expression via cDNA over-expression and shRNAs knock-down systems in prostate cancer cells, followed by examining NE marker expression and phenotypes of prostate cancer cells in *in vitro* (cell lines) and *in vivo*. Silencing REST in C4-2 (NE-/AR+) adenocarcinoma cells leads to the induction of NE marker SYP (Fig. 2A). Conversely, overexpressing REST in PC3 cells (NE+/AR-)^54^ downregulates mRNA (right) and protein levels (left) of NE markers (Fig. 2B). Phenotypically, REST overexpression dramatically reduced colony formation of PC3 cells (Fig. 2C). Moreover, REST silencing increased (Fig. 2D), while its overexpression decreased (Fig. 2E), the viability of LNCaP cells under treatment with ADT drug MDV3100 (Enzalutamide). To examine REST’s role in regulating prostate tumor growth in mouse xenografts, PC3-EV (empty vector) and PC3-REST cells were implanted subcutaneously (s.c.) in NOD/SCID male mice for 24 days. Xenograft tumors from PC3-REST cells are significantly smaller than tumors from PC3-EV cells (Fig. 2F). These results demonstrated that REST is a tumor suppressor gene in prostate cancer.

### ADT downregulates REST both in vitro and in vivo.

**Figure 3:**
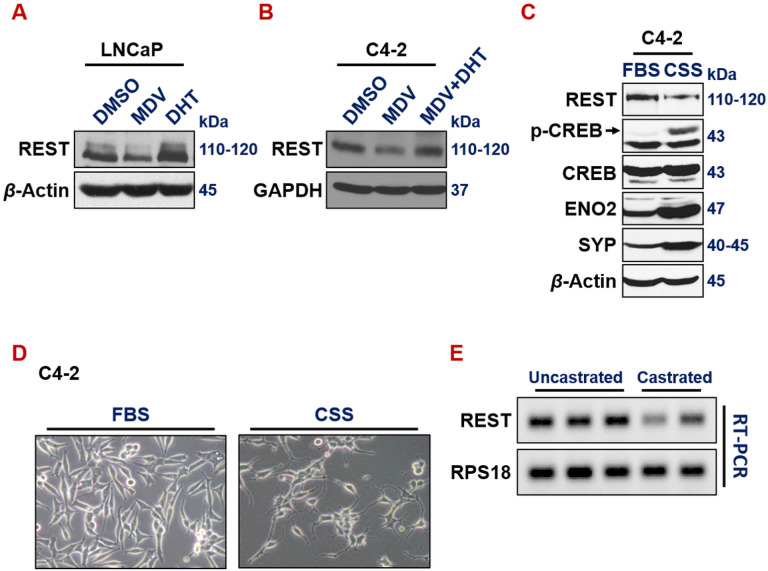
ADT downregulates REST *in vitro* and *in vivo*. (**A-B**) Western blotting of REST in LNCaP and C4-2 cells treated with ADT drug MDV3100 (10uM, 72 hours), androgen DHT (25nM, 24 hours) or both. (**C**) Western blotting of whole cell lysates from C4-2 cells growing in regular FBS or CSS (charcoal stripped serum) for 7 days. Culturing in CSS media that is deprived of hormones is another common ADT approach *in vitro*. (**D**)Morphology of C4-2 cells growing in regular FBS or CSS. (**E**) RT-PCR and DNA gel electrophoresis of REST and loading control RPS18 in LNCaP xenograft tumors growing in uncastrated or castrated NOD/SCID male mice.

We and others have reported that ADT promotes NE phenotypes in prostate cancer cells^3, 20, 55–59^. However, the exact mechanism underlying the induction of neuroendocrine differentiation by ADT is still incompletely understood. We speculated that ADT induces NE markers, at least in part, through downregulating REST, which is a well-established master repressor of NE phenotypes. Indeed, treatment of ADT drug MDV3100 in androgen-dependent LNCaP cells downregulates REST (Fig. 3A). Interestingly, treating LNCaP cells with androgen Dihydrotestosterone (DHT) induces REST (Fig. 3A). Similarly, REST is reduced by MDV3100 in CRPC cells C4-2, and this reduction was rescued by adding DHT along with MDV3100 (Fig. 3B). Culturing prostate cancer cells in media with charcoal-stripped serum (CSS), which is hormone deprived, is another common method to introduce ADT in culture. REST expression is also downregulated when C4-2 cells were cultured in media with CSS (Fig. 3C). Correspondingly, C4-2 cells exhibited cell morphology changes that are reminiscent of NE phenotypes, such as extended neurite spikes and size-reduced cell bodies (Fig. 3D). To examine the impact of ADT on REST expression *in vivo*, we carried out REST RT-PCR on LNCaP xenograft tumors growing in uncastrated vs castrated male NOD/SCID mice^20^. Castration, i.e. surgical removal of the major androgen-producing organ testis, is a common method of ADT *in vivo*. As shown in Fig. 3E, REST expression is downregulated by castration. Altogether, these results clearly show that ADT downregulates REST expression.

### Activated CREB signaling represses REST.

**Figure 4:**
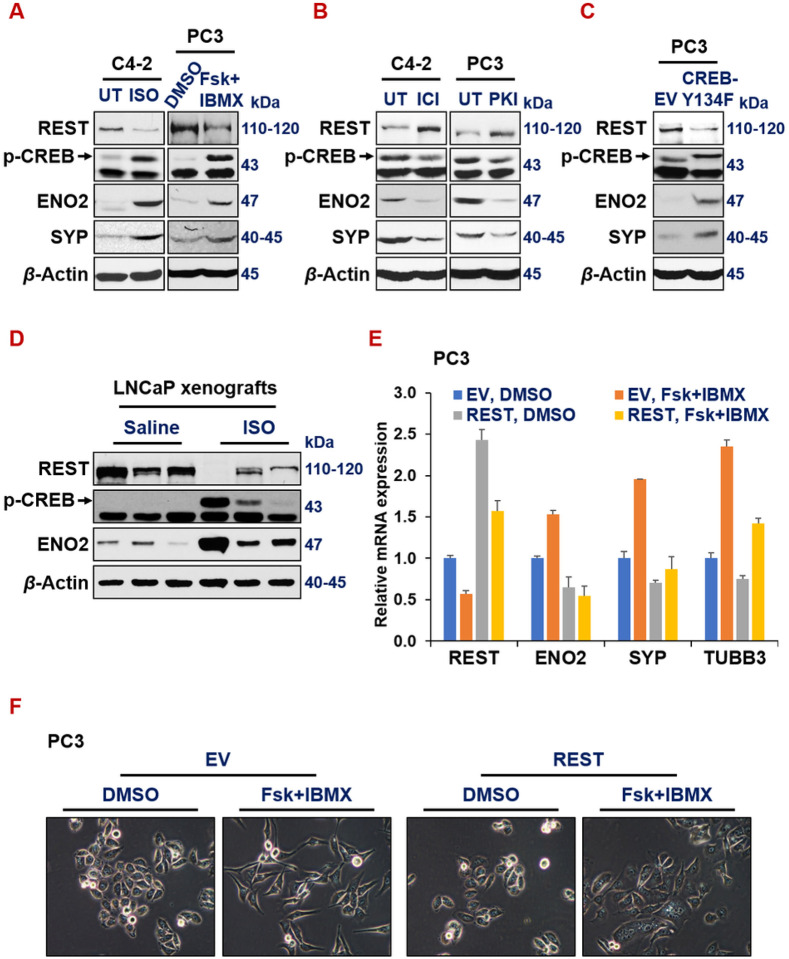
Activated CREB signaling represses REST. (**A-B**) Western blotting for REST, p-CREB1 (pS133, indicator of activation) and NE markers in prostate cancer cells (C4-2 on left and PC3 on right) upon treatments of CREB1 signaling activator 15uM isoproterenol (ISO) or 10uM Forskolin+ 0.5mM IBMX for 24hr (A), or CREB1 signaling inhibitor *ICI*-118,551 (*ICI, 10uM*) or synthetic peptide inhibitor of PKA (PKI, 10uM) for 24hr (B). (**C**) Western blotting for REST, pS133-CREB1 and NE markers in PC3 cells carrying either an empty vector or CREB1-Y134F cDNA, a constitutively activated form of CREB1. (**D**)Western blotting for REST, pS133-CREB1, NE markers, EZH2 catalytic product H3K27me3 histone mark, loading controls histone 3 (H3) and beta actin in LNCaP cell-derived xenografts from NOD/SCID male mice treated with saline or 10mg/kg ISO for 54 days. (**E-F**) PC3-EV or PC3-REST cells were treated with DMSO control or CREB1 signaling activator combo 10uM Forskolin (Fsk) + 0.5mM IBMX for 24hr. mRNA levels of indicted genes were normalized to GAPDH controls. (F) Morphology of PC3-EV and PC3-REST cells treated with DMSO or Fsk+IBMX.

We next investigated how ADT downregulates REST transcription in prostate cancer cells. CREB1 signaling and REST are known to antagonize each other in controlling neuronal gene expression in neurobiology^12–14^. Their relationship in prostate cancer cells is unknown. We previously showed that ADT activates CREB1 signaling, which is critical for NED of prostate cancer cells. We speculated that CREB1 signaling downregulates REST expression. To test this hypothesis, we first treated prostate cancer cells with known compounds that either enhance or inhibit CREB1 signaling, followed by examining REST and NE marker expression. Isoproterenol (ISO), an analog of adrenaline, acts as an agonist for beta-adrenergic receptor, enhancing PKA/CREB1 signaling^60, 61^. Additionally, the combination of Forskolin and IBMX (Fsk + IBMX) also activates PKA/CREB1 pathway^62^. Conversely, beta-adrenergic receptor antagonists ICI-118,551 (ICI) and synthetic peptide inhibitor of PKA (PKI) are two known inhibitors of PKA/CREB1 signaling. We found that REST levels are reduced, while p-S133-CREB1 (an indicator of CREB1 activation) is increased, when prostate cancer cells are treated with ISO or Fsk + IBMX (Fig. 4A, Supplementary Fig. S2A and S2B). On the contrary, REST is induced, and p-S133-CREB is reduced by ICI or PKI (Fig. 4B). When CREB1 signaling is enhanced by overexpressing a constitutively activated form of CREB1 cDNA (i.e. CREB1-Y134F^63^), REST is downregulated while NE markers ENO2 and CHGA are induced in PC3 cells (Fig. 4C and Supplementary Fig. S2C). To evaluate this CREB1-repression of REST *in vivo*, we measured protein levels of REST and ENO2 in LNCaP cell-derived xenografts from NOD/SCID male mice treated with saline or 10 mg/kg ISO twice a day for 21 days. As showed in Fig. 4D, REST is downregulated and NE marker ENO2 is induced in LNCaP tumors from the mice treated with ISO. Moreover, we found that REST cDNA overexpression in PC3 cells reversed NE marker induction by the activation of CREB1 signaling by Fsk + IBMX (Fig. 4E), which suggests that REST downregulation is essential for CREB1’s induction of NE markers in prostate cancer cells. Concordantly, the NE-like cell morphology induced by CREB1 activation was abrogated by REST overexpression in PC3 and LNCaP cells (Fig. 4F and Supplementary Fig. S2D respectively). All these results together indicate that CREB1 signaling induces NE markers through repressing REST expression in prostate cancer cells.

### REST is a novel epigenetic target of EZH2 and it reverses EZH2-induction of NE markers.

**Figure 5:**
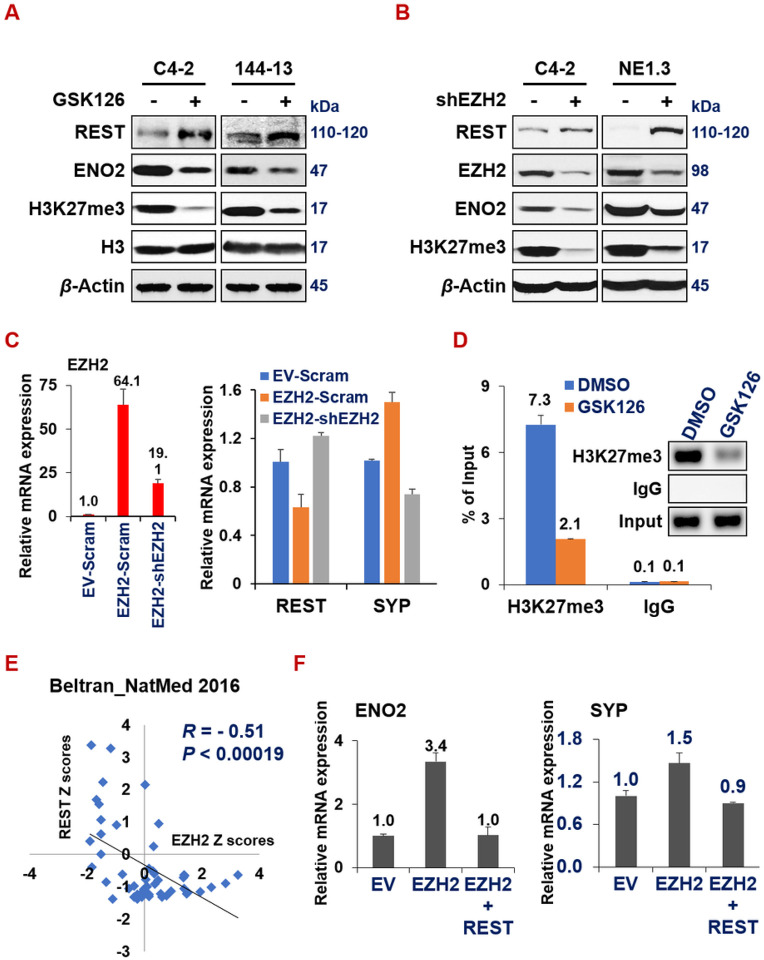
REST is a novel epigenetic target of EZH2 and it reverses EZH2-induction of NE markers. (**A**) C4-2 and 144-13 cells were treated with DMSO or 10uM GSK126 for 48hr. Whole cell lysate was analyzed by Western blotting. (**B**) Western blotting of indicated proteins in C4-2 and NE1.3 cells expressing scramble shRNA or shEZH2. (**C**) RT-qPCR showing that EZH2-mediated REST downregulation and SYP upregulation was reversed by shEZH2 (genetic rescue). (**D**) 144-13 NEPC cells were treated with DMSO or 10uM GSK126 for 48hr, followed by ChIP with control IgG or anti-H3K27me3 antibody, and qPCR of REST’s promoter region near its transcriptional starting site. Y-axis represents % of ChIPed DNA relative to input. (**E**) Scatter plots showing negative correlation of the expression of EZH2 and REST in the Beltran_NM2016 CRPC genomic dataset. The plot, Pearson correlation coefficient R and P value were directly downloaded from the cBioPortal genomics interface. (**F**)A genetic rescue experiment in LNCaP cells indicates that the induction of NE markers by EZH2 was reversed by additional overexpression of REST.

In the literature, there have been several elegant studies documenting the repression of REST function in prostate cancer through the mechanisms of alternative splicing and protein degradation^64–67^. The transcriptional regulation responsible for REST downregulation in NEPC is still obscure, despite the observation that REST mRNA level is pronouncedly reduced in NEPC (Fig. 1). Therefore, we considered that REST can be downregulated by transcriptional repressors, specifically epigenetic modulators. In a recent study surveying 147 epigenetic regulators in multiple patient datasets, Clermont *et al* found that several PRC2 complex proteins, such as EZH2 and CBX2, are among the most upregulated epigenetic regulators in NEPC^41^). We and others have recently implicated a role of EZH2 in regulating NE phenotype in prostate cancer cells^20, 39, 40, 68^. However, the mechanism underlying EZH2’s induction of NE markers is still unclear. We hypothesized that REST downregulation in NEPC is at least partially influenced by the increased activity of the PRC2 complex, and REST is a target of EZH2. Indeed, treating several prostate cancer cell lines with GSK126, an inhibitor of EZH2, resulted in REST induction and reduced NE marker ENO2 expression (Fig. 5A and Fig. S3A). Similarly, REST was induced and ENO2 was downregulated upon silencing EZH2 with shRNA (Fig. 5B). In line with these results, overexpressing EZH2 cDNA downregulated REST expression (Fig. 5C, left), and this downregulation was rescued by additional expression of shEZH2. Furthermore, NE maker SYP was induced by EZH2 cDNA, which was reversed by additional expression of shEZH2, as expected (Fig. 5C, right).

To determine whether REST is a direct target of EZH2, we first examined ENCODE ChIP-seq datasets. We found that EZH2 binds to REST promoter in multiple cell lines and under several conditions (Fig. S3B), which suggests that REST is an epigenetic target of EZH2. To confirm this in NEPC cells, we performed chromatin immunoprecipitation (ChIP) using antibodies against H3K27me3, a histone repressive mark primarily catalyzed by EZH2. We carried out H3K27me3 ChIP and qPCR of the DNA sequence on REST promotor in NEPC 144 − 13 cells treated with DMSO control or EZH2 inhibitor GSK126. As shown in Fig. 5D, there is a clear H3K27me3 mark on REST promoter in DMSO treated cells, which is reduced by GSK126. Consistently with REST being a novel EZH2-repressed target in NEPC, they have a significantly negative correlation of expression in two well-cited advanced prostate cancer datasets (Beltran_NatMed^39^ in Fig. 5E and SU2C-PCF^53^ in Supplementary Fig. S3C). Finally, we set out to determine whether REST downregulation is essential for EZH2’s induction of NE markers, by performing an epistasis experiment (genetic rescue). Of note, overexpression of REST reversed the induction of ENO2 and SYP by EZH2 overexpression in LNCaP cells (Fig. 5F). These results establish that REST is a novel epigenetic target of EZH2, whose repression is critical for EZH2’s induction of NE markers.

### ADT induces NE by downregulating REST through CREB-activated EZH2 epigenetic repression.

**Figure 6:**
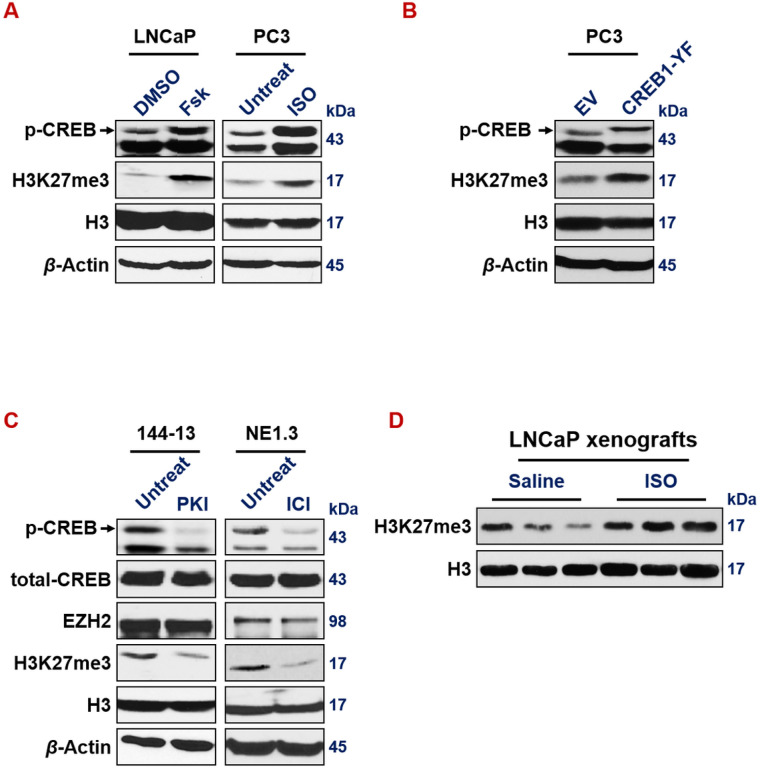
CREB1 signaling enhances EZH2’s PRC2 activity. (**A**) CREB1 signaling activator Fsk (left) (10um for 24hr in LNCaP cells) and with ISO (right) (15um for 24hr in PC3 cells) activates p-S133-CREB1 and induces bulk H3K27me3 levels. (**B**) p-S133-CREB1 and H3K27me3 levels are induced in PC3 cells expressing constitutively activated CREB1-Y134F mutant cDNA. (**C**) CREB1 signaling inhibitor PKI (left) (10um for 24hr in 144-13 cells) and with ICI (right) (10um for 24hr in NE1.3 cells) reduces p-S133-CREB1 and bulk H3K27me3 levels.

We previously reported that CREB1 signaling leads to the activation of EZH2 which subsequently induces NE markers^20^. Here we confirmed this conclusion, by showing that expressing activated CREB1 cDNA (Y134F), or treatment of CREB1 signaling activator Forskolin or ISO, increases H3K27me3 epigenetic mark in LNCaP and PC3 prostate cancer cells (Fig. 6A and 6B). Conversely, CREB1 signaling inhibitor PKI or ICI reduces H3K27me3 level in 144 − 13 and NE1.3 NEPC cells (Fig. 6C). ISO treatment also induces bulk level of H3K27me3 histone mark in LNCaP mouse xenograft tumors (Fig. 6D).

**Figure 7:**
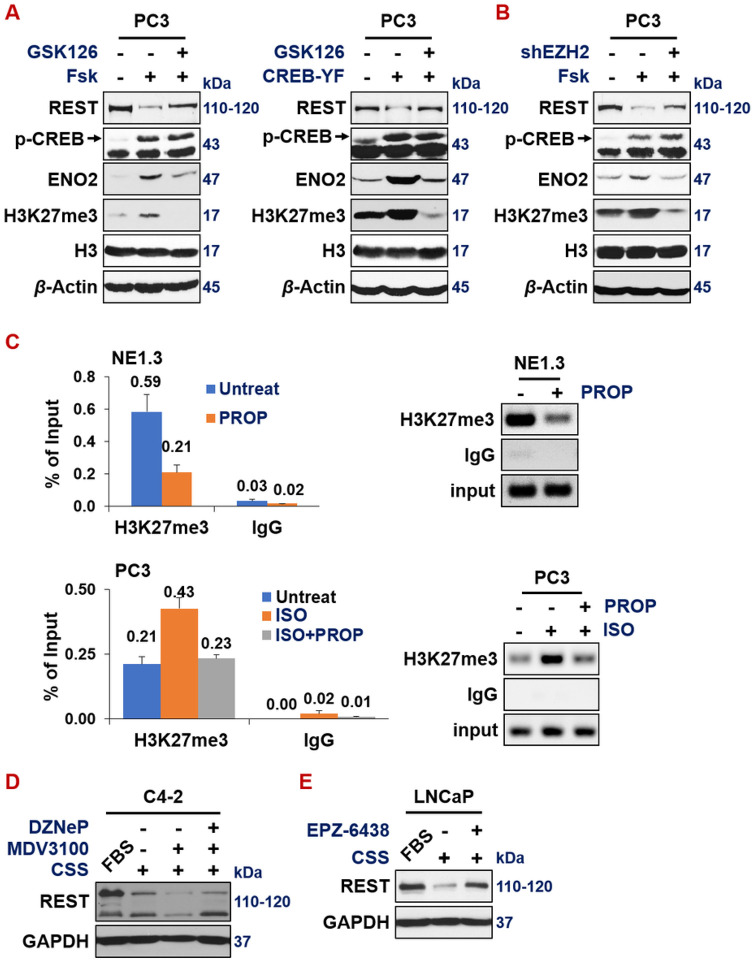
ADT induces NE by downregulating REST through CREB1-activated EZH2 epigenetic repression. (**A**) Western blotting on PC3 cells shows that REST is downregulated by CREB1 signaling activator forskolin (10uM, left) or by overexpressing activated CREB1 cDNA (right), which is reversed by EZH2 inhibitor GSK126 (10uM). (**B**) Western blotting shows that, when EZH2 is silenced by shEZH2, forskolin could no longer repress REST and induce NE marker ENO2 and H3K27me3 in PC3 cells. (**C**) ChIP-qPCR measuring H3K27me3 histone mark levels on REST promoter, after treating with indicated CREB1 signaling modulators: propranolol (PRO, 15uM) for 24hr in NE1.3 cells (left), or isoproterenol (ISO, 15uM) +/− propranolol (PRO, 15uM) for 24hr in PC3 cells (right). (**D**) PC3 cells were treated with DMSO, CREB1 signaling activator Forskolin (Fsk, 10uM) with or without EZH2 inhibitor GSK126 (10uM), for 24hr. Whole cell lysate and Western blotting indicate that REST repression by Fsk was rescued by GSK126. (**B**) PC3-shScramble control cells and shEZH2 cells were treated with DMSO or 10uM Fsk for 24hr, followed by Western blotting. (**C**) PC3-EV control cells and PC3 cells expressing CREB1-Y134F were treated with DMSO or 10uM GSK126 for 24hr, followed by Western blotting. (**D**)C4-2 cells were grown in FBS or CSS media for 7 days, then treated with DMSO or 10uM MDV3100 for 3 days, followed by treatments of DMSO or 5uM DZNeP for 2 days (as indicated). Whole cell lysates were collected and analyzed by western blotting for REST and loading control GAPDH.

Given that now we have illustrated that REST is a direct target of EZH2 in NEPC cells, next, we set out to determine the genetic relations of the three proteins on this CREB1-EZH2-REST pathway in the context of ADT-induction of NE markers in prostate cancer cells. First, repression of REST by CREB1 activators Forskolin + IBMX is reversed by EZH2 inhibitor GSK126 (Fig. 7A) or shEZH2 (Fig. 7B), suggesting that EZH2 is key to the CREB1-mediated repression of REST.

A similar result was observed when CREB1 signaling was activated by expressing constitutively active CREB1 cDNA (Y134F) (Fig. 7A). Second, as shown by ChIP-PCR, the H3K27me3 histone mark on REST promoter is evidently lower in NE1.3 cells treated with propranolol (PRO), another beta-adrenergic antagonist and an inhibitor of CREB1 signaling^20, 59, 69^. Accordantly, H3K27me3 histone mark on REST promoter is induced by CREB1 signaling activator ISO, which is reversed by additional treatment with PRO (Fig. 7C). Third, when EZH2 activity is inhibited by its inhibitor DZNeP in C4-2 cells, ADT, which is induced by growing cells in CSS media and treated with MDV300, no longer represses REST expression (Fig. 7D).

In summary, we have delineated a new critical pathway of CREB1-EZH2-REST, which underlies NE induction by ADT. We showed that REST is downregulated in NEPC cells, PDXs and patient samples. As expected, REST represses NE markers and prostate cancer progression. Furthermore, ADT downregulates REST both *in vitro* and *in vivo*. Nortably, ADT-activated CREB1 signaling enhances EZH2’s epigenetic repression of REST, which in turn induces NE markers in prostate cancer cells.

## Discussion

It has been showed by numerous studies that ADT induces NE markers in prostate cancer cells. However, the mechanisms underlying NE induction by ADT are incompletely understood. Here we first showed that ADT induces NE markers by downregulating REST, the master NE repressor. The majority of studies on the regulations of REST expression in cancer have been on its protein degradation or alternative splicing^64–67^. We found that REST mRNA level is pronouncedly reduced in NEPC (Fig. 1). The transcriptional regulation responsible for REST downregulation in NEPC is less clear. We further reported that REST is transcriptionally downregulated by EZH2, which is upregulated in NEPC and activated by CREB1 signaling.

REST and CREB1 have been reported to functionally antagonize each other in regulating neurogenesis^12–14^. For example, Laneve *et al* showed that CREB1 induces, while REST reduces, neuronal miRNA miR-9-2 in neuroblastoma cells^12^. However, as far as we know, a direct repression between these two master regulators has not been reported. Our study provides evidence that CREB1 signaling represses REST mRNA and protein expression during neuroendocrine differentiation of prostate cancer cells (Fig. 4). It is worth examining whether this CREB1 repression of REST transcription also occurs in neurobiology.

As to how CREB1 downregulates REST expression, we demonstrated that CREB1 signaling enhances EZH2 activity, which in turn epigenetically represses REST. Interestingly, Laneve *et al* further showed a negative feedback regulation between miR-9-2 and REST^12^. It warrants further investigation to determine whether miR-9-2 induction also contributes to REST repression by CREB1 signaling, besides EZH2’s epigenetic regulation which we demonstrated in this study.

Our study also reveals a surprising relationship between REST and EZH2. EZH2 and REST have been viewed as partners in transcriptional repression in several contexts. It has been reported that REST physically interact with PRC2 complex proteins EZH2 and SUZ12^48–50^. Furthermore, ENCODE ChIP datasets showed significant overlap of target genes between REST and EZH2 or SUZ12. In addition to recruitment by REST, the enzymatic PRC2 core component EZH2 can methylate REST, which leads to the stabilization of REST when bound to target gene RE1 sites^51^. Therefore, it is counterintuitive that EZH2 represses REST expression, as we have shown in Fig. 5. EZH2 and REST clearly have opposite functions and expression patterns in NEPC compared to prostate adenocarcinoma^20, 39, 40, 68^ (Figs. 1, 2 and 5). Therefore, our study introduces a conceptual innovation and context-dependence in the relationship between EZH2 and REST. It remains an interesting question for future investigation regarding how the EZH2-REST relationship evolves during prostate cancer progression, from REST being an EZH2 partner in prostate adenocarcinoma cells to REST becoming an EZH2 target in NEPC cells.

To summarize, we have demonstrated that REST is a novel epigenetically repressed target of EZH2 in NEPC and this EZH2-REST axis is essential for ADT-induced NED. Our results provide critical new insights into the mechanisms underlying NED that is induced by ADT and EZH2 activity. In addition, our findings have revealed a direct antagonistic relationship between two master regulators of neuronal genes in neurobiology, CREB1 and REST. These results not only expand our understanding of prostate cancer progression, they will also forge links among multiple disciplines, including cancer progression, drug resistance, cellular differentiation, epigenetic regulation, and neurobiology.

## Method

### Cell culture

LNCaP cells and 293T cells were originally purchased from ATCC. The PC3 prostate cancer cells used in this study represent a poorly metastatic PC3 variant that was kindly provided by Dr. Isaiah Fidler^70^ and was matched to PC3 cells from ATCC by DNA STR fingerprinting (Biosynthesis Inc). LNCaP and PC3 cells were maintained in RPMI 1640 media (Mediatech), supplemented with 10% FBS (Gibco) and 1% penicillin-streptomycin. 293T cells were cultured in DMEM media (Mediatech), supplemented with 10% FBS and 1% penicillin-streptomycin. NEPC NE1.3 cells were derived in Dr. Ming-Fong Lin’s lab from LNCaP cells after long term culturing in charcoal striped serum (CSS) medium^71^ and they are cultured in phenol red-free RPMI 1640 medium supplemented with 5% CSS (Gibco) and 1% penicillin and streptomycin. We generated LNCaP-AI (androgen independent) cells in house by culturing LNCaP cells in RPMI 1640 media with CSS for > 12 months and are maintained in the same CSS media. LN3 is a LNCaP derivative isolated from *in vivo* selection of LNCaP cells metastasizing to lymph nodes in NOD/SCID mice^70^. LNCaP, LNCaP-AI, LN3 and NE1.3 lines were all matched to ATCC LNCaP profile by DNA STR fingerprinting (Biosynthesis Inc). NEPC/SCPC cell line 144 − 13 were kindly provided by Drs. Ana Aparicio and Nora Navone and cultured as described^72^. Cultures were grown in a 37°C incubator with 5% CO_2_. All cell lines were routinely confirmed to be mycoplasma-free using the Lonza MycoAlert Detection kit (LT07-218).

#### In vitro treatments with activators and inhibitors

The activators and inhibitors used in this study were obtained from the following sources: Isoproterenol (ISO) (Sigma), Forskolin (FSK, LC Laborartoy), IBMX (Adipogen), ICI118,551 (ICI) (Tocris), propranolol (PRO) (Tci America), protein kinase A inhibitor peptide 14–22 (PKI) (Tocris), GSK126 (Selleck), MDV3100 (Apexbio) and Doxycycline (Enzo). The doses and duration of their treatments were as indicated.

### cDNA/shRNA transduction and transfection

All shRNA constructs are in pLKO.1 vector^73^ and were purchased from Sigma-Aldrich (St. Louis, MO). For stable knockdown, cells were transduced with lentiviral particles of Scramble control shRNA: CCTAAGGTTAAGTCGCCCTCG; EZH2 shRNA: (TRCN#40076) CGGAAATCTTAAACCAAGAAT^42, 74^; shREST: (TRCN#14785) GCCTCTAATCAACATGAAGTA. These shRNAs were packaged into viral particles using 293T cells according to previously described method^73^. Briefly, 293T cells were seeded in 6-well plates at 1.5-million cells/well. Lentiviral vector carrying shRNA was transfected, together with packaging plasmids VSVG and Delta 8.9, into 293 T cells by TransIT-LT1, followed by centrifugation at 1100xg for 30 min. After initial medium change around 16 hours post-transfection, the virus supernatant was collected 48 and 72 hours after transfection, aliquoted and stored at − 80°C for subsequent experiments. Cells were infected with the lentivirus supernatant in the presence of 8 μg/ml polybrene and subsequently selected with 1 μg/ml of puromycin. For overexpression of EZH2, cells were infected with retrovirus for human EZH2 cDNA or pBABE-puro vector control^75^, and subsequently selected with 1 μg/ml of puromycin. -For expressing constitutively active CREB1 mutant, PC3 cells were transfected with pcDNA3-Flag-empty vector (EV), or pcDNA3-CREB-Y134F (YF, constitutively active^63^), kindly provided by Dr. Rebecca Berdeaux, using TransIT-LT1 transfection reagent (Mirus) and selection with 400 μg/ml of G418 for 2 weeks. REST overexpression was achieved by LT1-mediated transfection of pcDNA3-EV, or pcDNA3-REST vector (a generous gift from Dr. Hsing-Jien Kung^18^) and selection with 400 μg/ml of G418 for 2 weeks.

### Reverse transcription and quantitative PCR (RT-qPCR)

Total RNA was extracted by using TRIzol Reagent (Life Technology). The RNA concentration and purity were measured by NanoDrop 2000 UV-Vis Spectrophotometer (Thermo Scientific). 2–3 μg of total RNA was used to generate cDNA using the iScript R Transcription Supermix (Bio-Rad). Real time qPCR was performed using SsoFast EvaGreen Supermix in CFX96 Thermal Cycler (Bio-Rad) or PowerUp SYBR Green Master Mix (Life Technology). PCR-based amplification was performed using the following primers:

REST F: tggaaaatgcaactatttttcaga; REST R: gaacttgagtaaggacaaagttcaca. EZH2 F: 5’-ccgctgaggatgtggatac-3’; EZH2 R: 5’-cagtgtgcagcccacaac-3’; CREB F: 5’-ggagcttgtaccaccggtaa-3’; CREB R: 5’-gcatctccactctgctggtt-3’; CHGA F: 5’-tacaaggagatccggaaagg-3’; CHGA R: 5’-ccatctcctcctcctcctct-3’; CHGB F: 5’-cacgccattctgagaagagc-3’; CHGB R: 5’-tctcctggctcttcaaggtg-3’; ENO2 F: 5’-ctgtggtggagcaagagaaa-3’; ENO2 R: 5’-acacccaggatggcattg-3’; SYP F: 5’-ccaatcagatgtagtctggtcagt-3’; SYP R: 5’-aggccttctcctgagctctt-3’. NTS F: 5’-gcatgctactcctggctttc-3’; NTS R: 5’-tggtcaagaaatctgcttctaatg-3’. TUBB3 F: 5’-atcagcaaggtgcgtgaggagtat-3’; TUBB3 R: 5’-tcgttgtcgatgcagtaggtc-3’. GAPDH F: 5’-agccacatcgctcagacac-3’; GAPDH R: 5’-gcccaatacgaccaaatcc-3’; Beta Actin F: 5’-ccaaccgcgagaagatga-3’; Beta Actin R: 5’-ccagaggcgtacagggatag-3’; RPS18 F: 5’-ctttgccatcactgccattaag-3’; RPS18 R: 5’-atcacacgttccacctcatc-3’. GAPDH or Beta Actin were used to normalize RNA input with similar results. The expression levels were calculated according to the comparative CT method (ΔΔCT).

### Western blotting analysis

Cells were washed in ice-cold PBS and lysed in lysis buffer (30 mM Tris, 200 mM NaCl, 1.5 mM MgCl_2_, 0.4 mM EDTA, 20% Glycerol, 1% NP-40, 1 mM DTT) with Complete mini protease inhibitor cocktail and PhosSTOP phosphatase inhibitor cocktail (Roche Applied Science). Protein concentrations were determined using Pierce BCA protein assay kit (Thermo Scientific). The samples were then separated by SDS-PAGE and transferred to PVDF membrane (Bio-Rad). The membrane was blocked with 5% skimmed milk in TBST for 1h at room temperature, followed by incubation of a primary antibody overnight at 4°C. The dilutions and catalog numbers of primary antibodies used are listed in Supplementary Table 1. After washes, the membrane was incubated with HRP-conjugated secondary antibodies for 1h at room temperature. The blots were then detected by Pierce ECL Western Blotting Substrate (Thermo Scientific) on Blue Basic Autoradiography Films (Thomas Scientific).

### Chromatin Immunoprecipitation (ChIP)

DNA binding proteins in cells were cross-linked to DNA by 1% formaldehyde for 10min at room temperature, which was quenched with glycine. Cells were then lysed in SDS Lysis Buffer (1% SDS, 10 mM EDTA, 50 mM Tris-HCl, pH 8.1 and freshly added protease/phosphatase inhibitors) and sonicated to shear DNA to 300–500 bp fragments using Branson Low Power Ultrasonic Systems 2000 LPt/LPe sonicator (Fisher Scientific). 50 μl of supernatant was diluted in 450 μl dilution buffer (1% Triton X-100, 2 mM EDTA, 20 mM Tris-HCl pH 8.1, 150 mM NaCl supplemented with 0.1% NP40, protease and phosphatase inhibitors). Samples were pre-cleared with protein A/G agrose beads for 2hr. 20 μl of the post-cleared supernatant was kept as input. The remaining supernatants were incubated overnight with 2 μg anti-H3K27me3 (Millipore) or anti-IgG antibody, followed by 1hr incubation with protein A/G agrose beads at 4°C. The immunoprecipitates were subjected to multiple washes for 5 minutes each at 4°C in low salt buffer with 150 mM NaCl, high salt buffer with 500 mM NaCl, LiCl buffer with 250 mM LiCl and finally the TE buffer. DNA was recovered after reversion of the protein-DNA cross-links with 0.2 M NaCl and proteinase K. Subsequently, DNA was extracted with phenol-chloroform and precipitated with ethanol. 5 μl of DNA was subjected to real time PCR. Primers used to measure the enrichment of REST promoter DNA sequence containing H3K27me3 marks are: F (5’-GTGGAAGGGTCTGAAATGGC-3’), and R (5’-GAACTCCCGACTTCTGGTGA-3’). The enrichment of ChIP DNA was calculated as percentage of input. The PCR products were resolved electrophoretically on a 2% agarose gel and visualized by ethidium bromide staining.

### Colony formation

Prostate cancer cells were seeded in a 6-well plate (1,000 cells/well) in RPMI 1640 media with 10% FBS and 0.5 mg/ml G418 for continuous selection. The cells were cultured for 14 days. Fresh media with G418 were replenished every 4 days. At day 14, cells were fixed with ice-cold methanol for 10 minutes and then stained with crystal violet solution for at least 30 min, followed by careful rinsing with sufficient ddH_2_O. After dry, image of the whole 6-well plate was taken.

### MDV3100 treatment and cell viability assay

Cancer cells were seeded in regular RPMI 1640 + 10% FBS media in a 96-well plate (quadruplicate or triplicate). Cells were treated with MDV3100 as indicated for 4 days. AlamarBlue reagent (Thermo Fisher Scientific) was then used to estimate cell numbers in the viability assays, as described^20, 59, 69, 76–78^.

### Mouse xenograft tumor experiments

All mouse studies followed protocols approved by the Animal Welfare Committee at the University of Texas Health Science Center at Houston. We have complied with all relevant ethical regulations. Male 6-7-week old NOD/SCID mice were implanted subcutaneously (s.c.) with two million of PC3-EV or PC3-REST cells in 100 μl 1:1 of PBS and Matrigel in both sides of each mouse (5 mice, n = 10 tumors for either group). The mice were sacrificed 8 weeks after the cell implantation. At sacrifice, the s.c. tumors were extracted, weighted and photographed. The tumors were then either formalin-fixed paraffin-embedded or snap-frozen.

### Genomics data mining

All non-TCGA and TCGA datasets indicated genes were downloaded from cBioPortal^79^ and the GEO database (http://www.ncbi.nlm.nih.gov/gds). The transformed and normalized gene expression values from these sources were used in our analysis and statistical calculation.

### Statistical analyses

Statistical analyses were performed using GraphPad software and/or online statistics tools. P values were obtained through Student t-test with two tails and unequal variance, unless otherwise indicated. Spearman correlation coefficient and associated P values for gene expression were calculated using GraphPad or a statistics tool at http://vassarstats.net/corr_rank.html and confirmed by another online tool: http://www.socscistatistics.com/tests/spearman/default2.aspx. P values < 0.05 are considered significant. All error bars are defined as s.d. All central values are defined as mean.

## Data Availability

The authors declare that the data supporting the findings of this study are available within the article and its supplementary information files, or are available upon reasonable requests to the authors.
